# Rest in Cardiac Affections

**Published:** 1894-04

**Authors:** 


REST IN CARDIAC AFFECTIONS.—Dr. T. Lauder Brunton (*The Practitioner*) believes that as a remedial measure, rest frequently requires to be absolute ; as a preventive one it may be relative. The amount enjoined must be carefully proportioned to each case, as in advanced mitral disease when the power of the heart is failing, absolute rest gives satisfactory results, in that the circulation recovers its balance, the arteries become filled and the veins emptied, the dropsical effusion and venous engorgement of the organs disappear, and the patient recovers a fair amount of health. In cases of mitral disease incompetence may come about from :

1. Enlargement of the auriculo-ventricular orifice.
2. Thickening of the valves, or
3. Incoördinate action of the musculi papillaries.

In the first case it may be hard to say if this be the sole cause of the regurgitation, without any obvious disease of the valve, as some disturbance of the relationship between the musculi papillaries may tend to aid the regurgitation. In such hearts in growing boys and in chlorotic girls, comparative rest may be useful, and sometimes absolute rest may be almost essential. In some cases the former may be all that is wanted as a prophylactic measure. In chlorotic girls gentle exercise is advisable, but it must be carefully graduated, as exhaustion is likely to do harm. Massage may be useful, as it gives the patient exercise without putting any strain upon the heart. With a fatty heart gentle exercise may be advisable, as it may be more likely to bring about a healthy condition of the heart than absolute rest. When in mitral disease cardiac tonics, even pushed to their utmost limit, fail to give relief, then absolute rest becomes of great importance. Massage is of great usefulness in clearing out the body-waste, quickening the flow of blood through the muscles, and relieving the œdema, and the patient gets the advantage of the exercise without overdoing his heart. It also allows the treatment to be carried out more easily than it would otherwise be, for it removes the feeling of weariness and irritability, faintness and unrest of the patient.—*Medical Review. Buffalo Medical and Surgical Journal.*

